# Discriminating Central Lung Cancer Tumors from Atelectasis using Radiomics Analysis on Contrast-free CT

**DOI:** 10.2174/0115734056348733250324234338

**Published:** 2025-04-30

**Authors:** Xiaoli Hu, Qianbiao Gu, Qian Guo, Feng Wu, Yinqi Liu, Zhuo He, Hongrong Shen, Kun Zhang

**Affiliations:** 1 Department of Radiology, First Affiliated Hospital of Hunan University of Chinese Medicine, Changsha, China; 2 Department of Radiology, Hunan Provincial People's Hospital, First Affiliated Hospital of Hunan Normal University, Changsha, China

**Keywords:** Central lung cancer, Atelectasis, Computed tomography, Radiomics, Decision curve analysis, Magnetic resonance imaging

## Abstract

**Background:**

Accurate determination of tumor boundaries is crucial for staging and treating central lung cancer (CLC).

**Objective:**

This retrospective study aimed to evaluate the feasibility of contrast-free CT radiomics in discriminating CLC tumors from atelectasis.

**Methods:**

A total of 58 patients with CLC and associated lung atelectasis, corresponding to 58 tumors and 58 atelectasis regions, were included. Radiomics features were extracted from tumor and atelectasis areas using contrast-free CT images. The least absolute shrinkage and selection operator (LASSO) identified the most differential radiomics features. A logistic regression model (LR) was established and evaluated using 5-fold cross-validation. Discrimination performance was assessed using the area under the ROC curve (AUC) and decision curve analysis (DCA). Additionally, the potential of visualizing and distinguishing tumors and atelectasis based on contrast-free CT was explored by comparing pixel-level radiomics features with contrast CT.

**Results:**

A total of 1561 radiomics features were extracted, with 356 showing significant statistical differences between tumor and atelectasis. LASSO identified the 10 most differential radiomics features. The LR model trained with these features achieved an AUC of 0.94 (95% CI: 0.89-0.99), sensitivity of 0.88, and specificity of 0.89 in the training group, and an AUC of 0.81 (95% CI: 0.67–0.95), sensitivity of 0.78, and specificity of 0.65 in the validation group. DCA confirmed the clinical utility, and the radiomics feature square_firstorder_10Percentile showed good performance in distinguishing tumors from atelectasis, with consistency to contrast CT.

**Conclusion:**

Contrast-free CT radiomics can effectively discriminate CLC tumors from atelectasis.

## 1 INTRODUCTION

Lung cancer is the leading cause of cancer-related deaths globally [1, 2]. Central lung cancer (CLC) is defined as a tumor originating from the bronchial lumen or wall, typically occurring in the segmental or more proximal bronchi [3]. It often presents with accompanying obstructive atelectasis. Accurate determination of tumor boundaries is crucial for tumor staging, assessability for resection, treatment evaluation, and radiotherapy target delineation [4-7]. Currently, computed tomography (CT) is the standard imaging modality for clinical diagnosis and management of lung cancer [8]. However, in conventional CT scans, obstructive atelectasis and tumor both appear as dense opacities, making it difficult to differentiate. Contrast CT has limitations such as additional radiation exposure and adverse contrast agent reactions [9-11]. Magnetic resonance imaging (MRI) and positron emission tomography/computed tomography (PET-CT) are widely used for tumor diagnosis, but they also have limitations such as susceptibility to motion artifacts, contraindications for MRI, high cost, and low availability of PET-CT.

Radiomics, a field of study in medical imaging, aims to extract and mine features from medical images noninvasively, enabling quantitative characterization of tissue heterogeneity. It has shown great potential in tumor diagnosis and prognosis assessment [12-16]. However, there is limited research on its ability to distinguish between tumor and atelectasis in CLC. Theoretically, tumors and atelectasis in CLC are two fundamentally different tissues. Tumors are characterized by unlimited replication, proliferation, and high metabolic activity [17], whereas atelectasis represents collapsed normal lung tissue. These differences lead to significant distinctions in cellular density, microenvironment, and blood supply between the two, which form the basis for differentiation using MRI-DWI sequences and PET-CT imaging [18]. Radiomics, by analyzing pixel intensity and its spatial distribution patterns in medical images, can further capture the microscopic differences between tumors and atelectasis [19]. Specifically, tumors typically exhibit heterogeneous pixel intensity distributions, reflecting the high heterogeneity within the tissue, such as high cellular density, active metabolism, and irregular neovascularization. These characteristics often result in tumor regions displaying more complex grayscale distribution patterns and spatial structures in medical images. In contrast, atelectasis, as collapsed normal lung tissue, is relatively homogeneous, lacking the heterogeneity and metabolic characteristics of tumors. Consequently, its pixel intensity distribution is more uniform, and its spatial distribution patterns are relatively simple. By quantitatively analyzing these pixel intensities and spatial distribution patterns, radiomics can extract high-dimensional features, such as texture features, gray-level co-occurrence matrix (GLCM), and gradient distribution. These features enable further quantification of the underlying differences between tumors and atelectasis, providing a robust basis for distinguishing between the two.

In this study, our objective is to explore the feasibility of using conventional contrast-free CT and radiomics methods to differentiate between CLC tumors and atelectasis. Then, to visualize pixel-level radiomic features on contrast-free CT to distinguish between tumor and atelectasis. We hope to provide a reliable and cost-effective approach to enhance the accurate characterization of CLC tumors and positively impact clinical decision-making and treatment outcomes.

## 2 MATERIALS AND METHODS

### 2.1 Patient Data

This study was conducted under the Helsinki Declaration (revised in 2013) and was approved by the ethics committee of the First Affiliated Hospital of Hunan University of Chinese Medicine. A retrospective collection of lung cancer patients confirmed by pathology from June 2020 to August 2023 was performed. Inclusion criteria were as follows: (1) CLC diagnosed according to established criteria [8], and (2) preoperative CT scan including both contrast-free CT and contrast CT images showing a tumor with associated lung atelectasis. Exclusion criteria were: (1) incomplete or obvious artifacts in the images, (2) prior anti-tumor therapy administration.

### 2.2 CT Image Acquisition

The GE Revolution 256 CT scanner (Revolution CT, GE Company, America) was used for performing whole-lung non-enhanced and contrast-enhanced scans. Patients were positioned in a supine position with the midline of the chest perpendicular to the scanning bed and aligned with the long axis of the bed. Both upper limbs were naturally raised to support the head. Scans were acquired in the head-to-toe direction, covering the range from the thoracic inlet to the lung base. Image acquisition was performed during breath-holding at the end of a full inspiration. The scan parameters were as follows: tube voltage 100-120 kV, tube current modulation 70-300 mAs, slice thickness 5 mm, 1 mm reconstructed slice thickness, 1 mm reconstructed slice gap, and a matrix of 512×512. For contrast-enhanced scans, intravenous injection of the contrast agent iopromide (350 mgI/mL) was administered at a flow rate of 2.5-3 mL/s, followed by arterial and venous phase acquisitions at 25 and 40 seconds after injection, respectively.

### 2.3 Annotation of the Regions of Interest (ROIs)

Two trained radiologists with over 10 years of experience used 3D Slicer software (version 5.2.1, https://www.slicer.org/) to place the ROIs after real-time consultation and reach a consensus. Circular ROIs with an area of 10-20 mm2 were selected. Referring to the corresponding contrast images, the ROIs were placed on the contrast-free CT axial images in the areas most confidently identified as tumor or atelectasis, while avoiding blood vessels and areas of cystic necrosis. The software interface of 3D Slicer and the placement of ROIs are illustrated in Fig. (**1**).

### 2.4 CT Radiomics Feature Extraction

Based on the 3D Slicer platform, the open-source radiomics package pyradiomics version 3.0.1 was utilized to extract radiomic features from the ROIs of the patients. The extraction of radiomic features in radiomics can be understood as a two-step process involving image transformation and feature calculation. The purpose of the image transformation step is to construct feature maps that are non-linearly correlated with the original images. In the feature calculation step, statistical and texture features are computed on both the original and transformed images. Extracting features from the transformed images increases the likelihood of obtaining valuable features for radiomics analysis. The image transformation techniques include built-in filters such as gradient, exponential, logarithmic, square root, wavelet, and logarithmic filters, which are applied to CT images to produce derived images. Ultimately, a total of 1561 radiomic features were extracted from each ROI.

### 2.5 Feature Selection and Radiomics Model Construction

We employed a three-step procedure to select the most informative radiomic features. Firstly, data preprocessing was performed using the Caret package in R software. This involved several specific steps: the nearZeroVar function was applied to remove features with zero or nearly zero variance. Then, the Wilcoxon rank-sum test or independent samples t-test was utilized to conduct preliminary analysis on the radiomic features between the tumor and lung atelectasis groups, identifying the features with significant differences between the two groups. Finally, LASSO regression was employed to determine the most distinguishing radiomic features and build a binary logistic regression model. The binary logistic regression model incorporated the 10 most discriminative radiomic features identified through LASSO feature selection. The model is specifically formulated as:

Here, P(Y=1) denotes the probability of the tumor, β0 is the intercept, β1, β2..., β10 are the regression coefficients, and X1, X2..., and X3 represent the selected radiomic features. The model's performance was evaluated using five-fold cross-validation.

### 2.6 Statistical Analysis

Statistical analyses were conducted using IBM SPSS Statistics software (version 25) or R software (version 3.6.0; http://www.r-project.org). Continuous variables were presented as mean ± standard deviation. For continuous variables, we first assessed the normality of distribution using the Shapiro-Wilk test. Normally distributed continuous variables were compared using the independent samples t-test, provided the assumption of homogeneity of variances was met, as confirmed by Levene's test. In cases where the assumption of homogeneity of variances was violated, Welch's t-test was utilized to accommodate unequal variances. For non-normally distributed continuous variables, the Wilcoxon rank-sum test was employed to ensure robust comparisons without the assumption of normality. Categorical data were expressed as frequencies and percentages. The multivariable binary logistic regression model was constructed using the “glmnet” package in R. This package is specifically designed for fitting regularized regression models, such as LASSO. The “glmnet” package offers computational efficiency through cyclical coordinate descent, flexibility in supporting logistic regression for binary outcomes, and automatic computation of regularization paths for model selection [20]. The area under the ROC curve (AUC), sensitivity, and specificity were used to assess the predictive ability of the model. Decision curves were employed to evaluate the clinical utility of the model.

## 3 RESULT

### 3.1 Patient Baseline Clinical Characteristics

A total of 58 patients with confirmed central-type lung cancer were included in this study, comprising 17 females and 41 males. The age of the patients ranged from 40 to 86 years, with a mean age of 64 ± 10 years. All patients presented with associated obstructive lung atelectasis. Among the cases, 39 were diagnosed through bronchoscopy, 10 through surgical confirmation, and 9 through cytological examination of pleural fluid. The pathological subtype distribution comprised 24 cases of squamous cell carcinoma, 25 cases of adenocarcinoma, 6 cases of small cell carcinoma, and 3 cases of other malignant tumors Table **1**.

### 3.2 Radiomic Features Differentiating CLC Tumors from Atelectasis

**Fig. (2):**
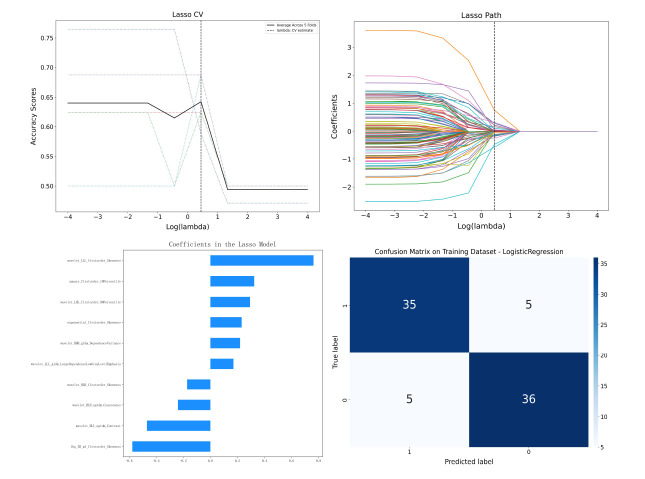
Least absolute shrinkage and selection operator (LASSO) feature selection and model performance. (**a**) LASSO cross-validation curve showing accuracy scores across 5-fold cross-validation for different λ values. The optimal λ (vertical dashed line) minimizes cross-validated error and determines the most predictive features. (**b**) Coefficient paths for 1561 radiomic features as a function of λ (log scale). At the optimal λ, 10 features with non-zero coefficients were retained. (**c**) Coefficients of the 10 selected features, ranked by their contribution to the logistic regression model. (**d**) Confusion matrix of the logistic regression model on the training dataset.

**Fig. (3):**
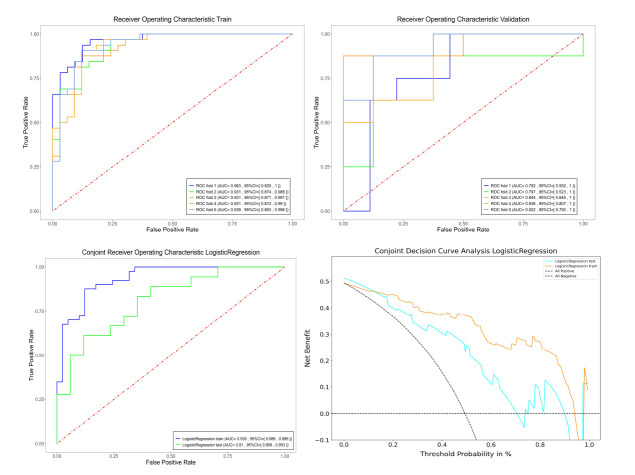
Performance and clinical utility of the Logistic Regression model. (**a**) ROC curves and AUCs for the training sets in 5-fold cross-validation, showing consistent performance. (**b**) ROC curves and AUCs for the validation sets in 5-fold cross-validation, demonstrating good generalizability. (**c**) Combined ROC curves for the entire training group and test group, illustrating robust predictive performance. (**d**) Decision curve analysis showing that the Logistic Regression model provides greater net benefit than "All Positive" or "All Negative" strategies across a range of threshold probabilities in both the training and test groups.

A total of 1561 radiomic features were extracted, and after preliminary analysis, 356 features were identified with significant statistical differences between the tumor and lung atelectasis groups. Following LASSO feature selection, a final set of 10 differentiating radiomic features was identified (Fig. **2**). The logistic regression (LR) model based on these features displayed an area under the receiver operating characteristic curve (ROC-AUC) of 0.94 (95% CI: 0.89-0.99) in the training group and 0.81 (95% CI: 0.67-0.95) in the validation group. Decision curve analysis (DCA) confirmed the clinical utility of the model (Fig. **3** and Table **2**).

### 3.3 Visualization of Radiomic Features for CLC Tumors and Atelectasis

**Fig. (4):**
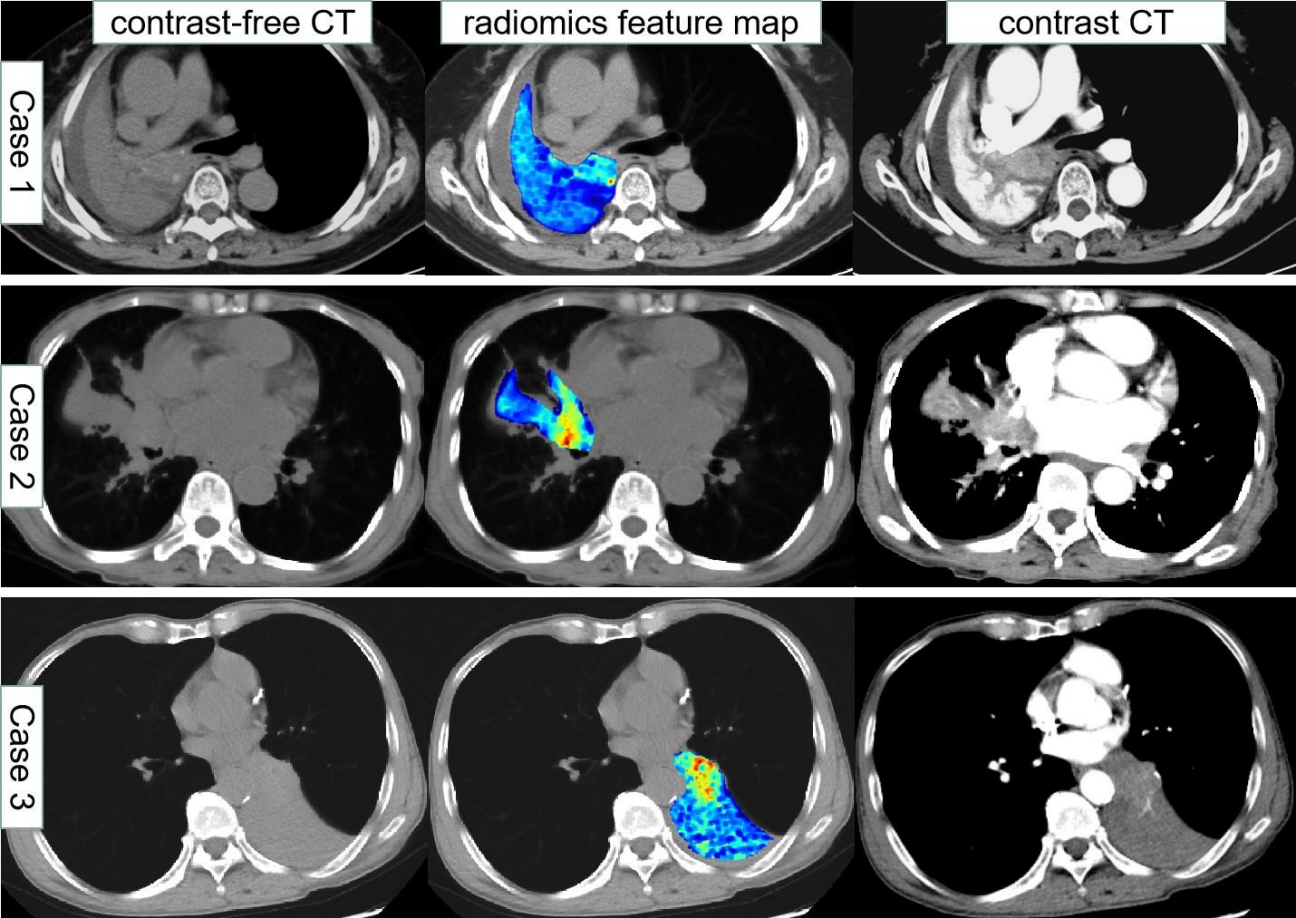
Visualization of radiomic features square_firstorder_10Percentile and comparison with contrast-free CT and contrast-enhanced CT images for three representative patients. The input images are axial slices of contrast-free CT scans focused on the lesion's largest cross-sectional area. Radiomic feature values for square_firstorder_10Percentile were calculated at the pixel level to generate radiomic feature maps, which were compared with the corresponding contrast-enhanced CT images. The radiomic feature maps reveal the distinct spatial distribution of square_firstorder_10Percentile, highlighting areas with significant differences between tumor and atelectasis.

**Fig. (5):**
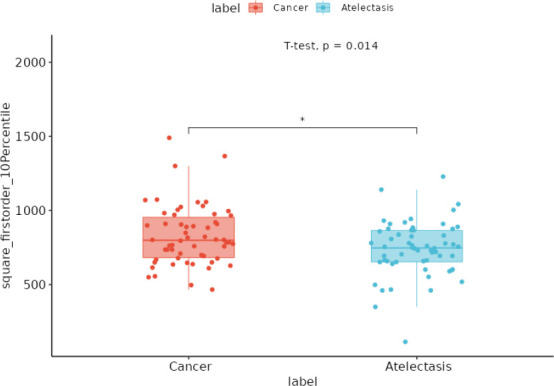
Value of “square_firstorder_10Percentile" between tumor and atelectasis. The feature value of "square_firstorder_10Percentile" in tumor was significantly higher than atelectasis.

The selected radiomic features were visualized at the pixel level and compared with the corresponding contrast CT images. During the visualization process, axial slices of contrast-free CT scans were selected based on the largest cross-sectional area of the lesion. Using PyRadiomics (voxel-based extraction), pixel-level square_firstorder_10Percentile values were calculated within the region of interest. The radiomic feature “square_firstorder_10Percentile” demonstrated good differentiation between tumors and atelectasis and showed consistency with the contrast CT images (Fig. **4**). Among the 58 cases, the “square_firstorder_10Percentile” radiomic feature successfully distinguished tumors from lung atelectasis in 38 cases (65.52%). Notably, the feature values of “square_firstorder_10Percentile” were significantly higher in tumors than in atelectasis (Fig. **5**). Compared to contrast-enhanced CT, the radiomic feature “square_firstorder_10Per centile” exhibited strong discriminative power in tumors with pronounced enhancement (30/35 cases). However, its performance was less stable in tumors with low enhancement (8/23 cases). Other radiomic features incorporated into the model, while demonstrating predictive efficacy within the integrated model, individually showed suboptimal visualization outcomes with accuracies all below 50%.

## 4 DISCUSSION

In this study, we investigated the application value of contrast-free CT-based radiomics analysis in differentiating central lung cancer (CLC) from atelectasis. We extracted a large number of radiomic features from contrast-free conventional CT scans and analyzed and evaluated these features using statistical methods. Firstly, our findings demonstrated the existence of numerous radiomic features with significant differences between tumors and atelectasis, which is consistent with the research by Chai *et al*. [21]. They found that both 2D and 3D radiomic features extracted from CT images could effectively differentiate lung cancer from atelectasis, but their study utilized contrast CT images. Our study, on the other hand, indicated that radiomics analysis based on contrast-free conventional CT images could also be used to distinguish lung cancer from atelectasis, avoiding additional radiation exposure and contrast agent-related adverse events.

Furthermore, based on the radiomic features finally selected by LASSO, our binary logistic regression model achieved an accuracy of 71.43%. This further corroborates the feasibility of using machine learning algorithms to segment tumors and atelectasis on contrast-free conventional CT images. Currently, there is limited research on differentiating lung cancer from atelectasis based on CT radiomics, with most studies focusing on magnetic resonance imaging. Studies by Yang *et al*. [22]. and Zhang *et al*. [23]. demonstrated that diffusion-weighted imaging (DWI) techniques can significantly enhance the visibility of lung cancer and atelectasis by improving the image signal-to-noise ratio compared to conventional MRI imaging. Lei and liang *et al*. [24, 25]. utilized contrast ultrasound and found that it could effectively differentiate lung cancer from atelectasis, providing valuable clinical guidance for puncture biopsy in CLC with associated atelectasis. However, in clinical practice, particularly in delineating the radiation target area for CLC, CT remains the routine and widespread method. Wen *et al*..’s research showed that neither conventional CT scans nor spectral CT images could accurately identify tumor boundaries in CLC [26]. Our study presented a new approach using radiomics analysis and the integration of multiple CT scan radiomic features, which effectively distinguished lung cancer from atelectasis, providing a new perspective for future research.

Additionally, through pixel-level visualization analysis of the selected radiomic features, we successfully differentiated tumors from atelectasis in 38 out of 58 cases (65.52%) using the radiomic feature of “square_firstorder_10Percentile”. This accuracy is comparable to the accuracy reported by Wen *et al*. [26]. based on spectral CT 40Kev monochromatic images (68.63%). “Square_firstorder_10Percentile” is a histogram feature that reflects the distribution characteristics of intensity signals in each pixel within the ROI. According to our findings, the square_firstorder_10Percentile value is significantly higher in tumors compared to atelectasis, likely due to the higher cellular density and heterogeneity of tumor tissue. These properties may result in restricted diffusion observed in diffusion-weighted imaging (DWI) studies [27, 28], reflecting underlying pathophysiological differences, such as increased tumor cell proliferation and altered stromal composition. While our study provides quantitative evidence supporting the differentiation between tumors and atelectasis, further validation of this feature's biological significance through additional modalities, such as histopathology or molecular analysis, will be essential. These efforts will help build a stronger theoretical foundation for understanding why square_firstorder_10Percentile effectively distinguishes central lung cancer tumors from atelectasis, thereby enhancing its clinical applicability. Contrast-enhanced CT primarily relies on vascular enhancement differences, providing direct visual cues for distinguishing tumors from atelectasis. The radiomic feature “square_firstorder_10Percentile” demonstrated strong discriminative power in distinguishing highly enhancing tumors from atelectasis (30/35 cases), likely due to the higher cellular density and greater heterogeneity of highly enhancing tumors. However, in low-contrast scenarios, such as cases of low tumor enhancement, radiomic features derived from non-contrast CT may face limitations, including boundary blurring and overlapping pixel intensities, which can reduce diagnostic accuracy. To address these challenges, future studies should integrate multiple radiomic features or incorporate deep learning models to enhance the classification performance and clinical utility of non-contrast CT.

There are several limitations to our study. Firstly, we excluded CLC patients whose primary tumors were still indistinct on contrast-enhanced CT scans, as we used the contrast CT images as a reference and placed the ROI on the non-enhanced CT scans. Thus, the potential application value of radiomics analysis in this subgroup warrants further investigation. Secondly, although we identified a set of differential radiomic features in this study, there are still many other features worth exploring. Future research could explore more sophisticated machine learning methodologies and deep learning architectures to uncover additional potentially discriminative features from complex and multidimensional imaging data. Lastly, the sample size in this study was relatively small, which may introduce sample bias. In future studies, it is necessary to expand the sample size with larger, multi-center datasets to assess the generalizability and clinical utility of the model. These steps will help address the identified limitations and further establish the clinical applicability of our findings.

## CONCLUSION

In this study, we employed contrast-free CT radiomics and feature visualization analysis to successfully differentiate between tumor and atelectasis in patients with CLC, providing a novel, cost-effective approach for clinical diagnosis and treatment of CLC. We identified 10 radiomic features with the highest discriminatory power, and the model we developed achieved AUC values of 0.94 in the training set and 0.81 in the validation set. Despite the small sample size and the exclusion of some cases, our method significantly reduces the use of contrast agents and decreases radiation exposure. It is especially useful for assisting in the T-staging of CLC in patients who cannot use iodine contrast agents and for more precisely delineating tumor target areas in radiation therapy. Future research should expand this approach through multicenter data and explore more advanced machine learning and deep learning technologies to further validate and optimize the model, ensuring its applicability and stability across various medical settings and promoting its widespread use in clinical practice..

## Figures and Tables

**Fig. (1):**
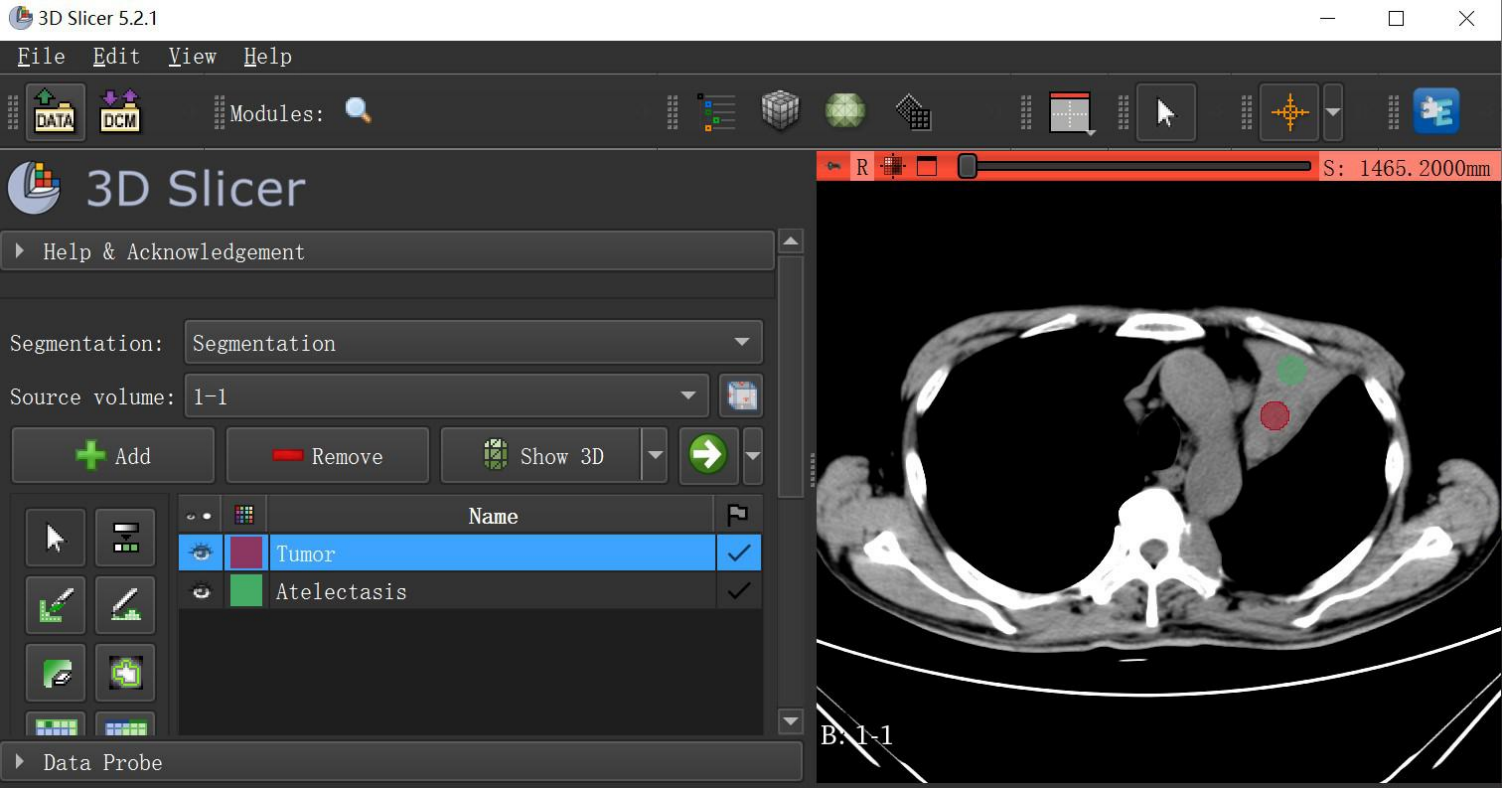
Interface for drawing rois in 3Dslicer software.

**Table 1:** The basic clinical and pathological characteristics of the research cohort.

**Characteristics**	**Total (N=58)**
Age (years) ^*^	64 ± 10
Gender (Male/Female)	41/17
Pathological subtype	-
Squamous cell carcinoma	24 (41.38)
Adenocarcinoma	25 (43.11)
Small cell carcinoma	6 (10.34)
Others	3 (5.17)
Diagnosis method	-
Bronchoscopy	39(67.24)
Surgery	10(17.24)
cytological examination of pleural fluid	9(15.52)

**Note: **Unless otherwise specified, data is presented as the number of patients (percentage); ^* ^Data shown as mean ± standard deviation.

**Table 2:** Prediction Performance of the model.

**Model**	**AUC**	**SEN**	**SPE**	**ACC**	**PPV**	**NPV**
LogisticRegression	-	-	-	-	-	-
Training Cohort	0.94	0.88	0.89	0.88	0.88	0.88
Validation Cohort	0.81	0.78	0.65	0.71	0.70	0.73

**Note: **AUC, area under the receiver operating characteristic curve; SEN, sensitivity; SPE, specificity; ACC, accuracy ; PPV, positive predictive value; NPV, negative predictive value.

## Data Availability

The datasets used and/or analyzed during the current study are available from the corresponding author [K.Z] upon reasonable request.

## LIST OF ABBREVIATIONS

CLC: Central lung cancer
LR: Logistic regression model
LASSO: Least absolute shrinkage and selection operator
ROC: Receiver operating characteristic curve
AUC: Area under the receiver operating characteristic curve
DCA: Decision curve analysis
CI: Confidence interval
CT: Computed tomography
MRI: Magnetic resonance imaging
ROI: Regions of interest
DWI: Diffusion-weighted imaging

## STANDARDS OF REPORTING

STROBE guidelines were followed
